# Supplementary figures and images for: Proteostasis is adaptive: Balancing chaperone holdases against foldases
Source: PLoS Comput Biol. 2020 Dec 14;16(12):e1008460. doi: 10.1371/journal.pcbi.1008460 (PMC7769611; doi:10.1371/journal.pcbi.1008460)

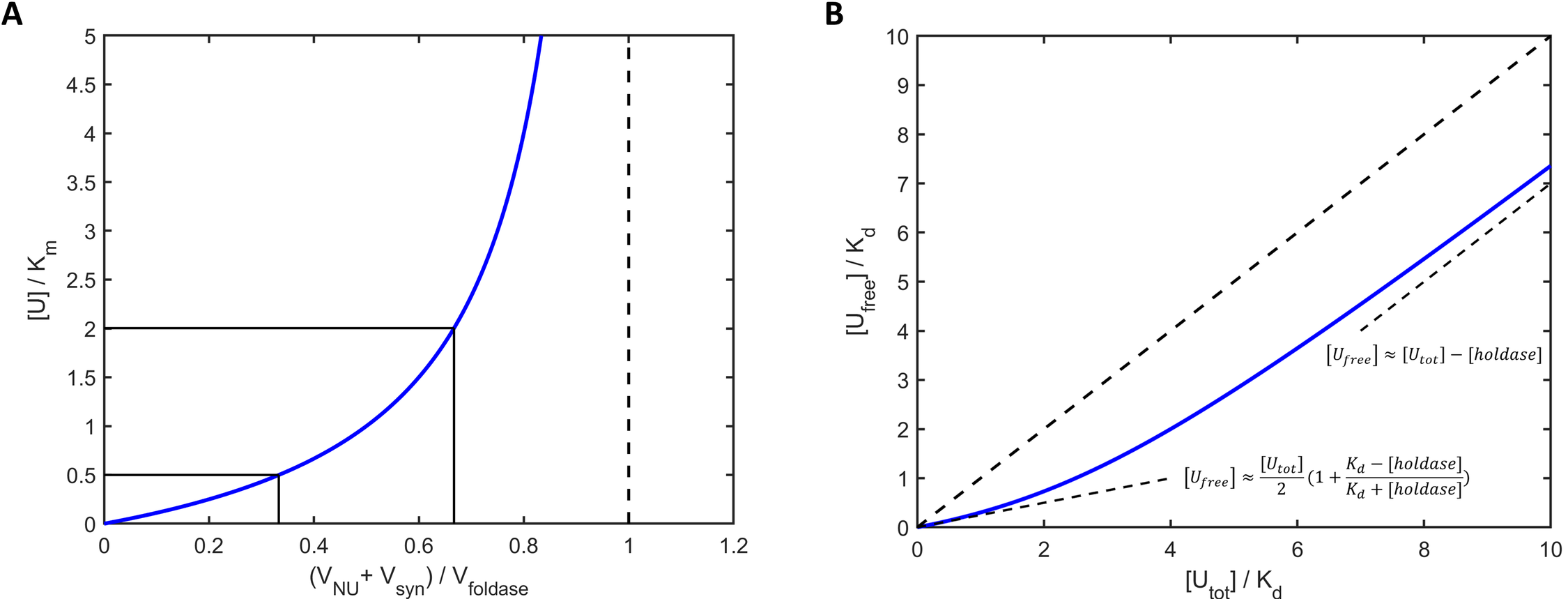

Supplement: S1 Fig — (A) Foldases actively promote the folding of proteins that are newly unfolded (flux vN→U) or newly synthesized (vsyn). The total foldase capacity (vfoldase) needs to be greater than the unfolding burden (vN→U + vsyn) to prevent unfolded levels from diverging (vertical dashed line is the limit of the Michaelis-Menten function describing foldase activity in blue). The benefit of a large spare foldase capacity can be seen from the rapid rise of the unfolded level [U], up 4-fold for only a 2-fold increase in unfolding load (thin black lines). (B) Holdases further deplete the level of unfolded protein by acting as protective “parking spots” while their unfolded clients wait for a foldase. The degree of depletion, given by the distance of the curve below the diagonal, can be solved in limiting cases (thin dashed lines, see Methods for the derivation). (TIF) [file pcbi.1008460.s001.tif]

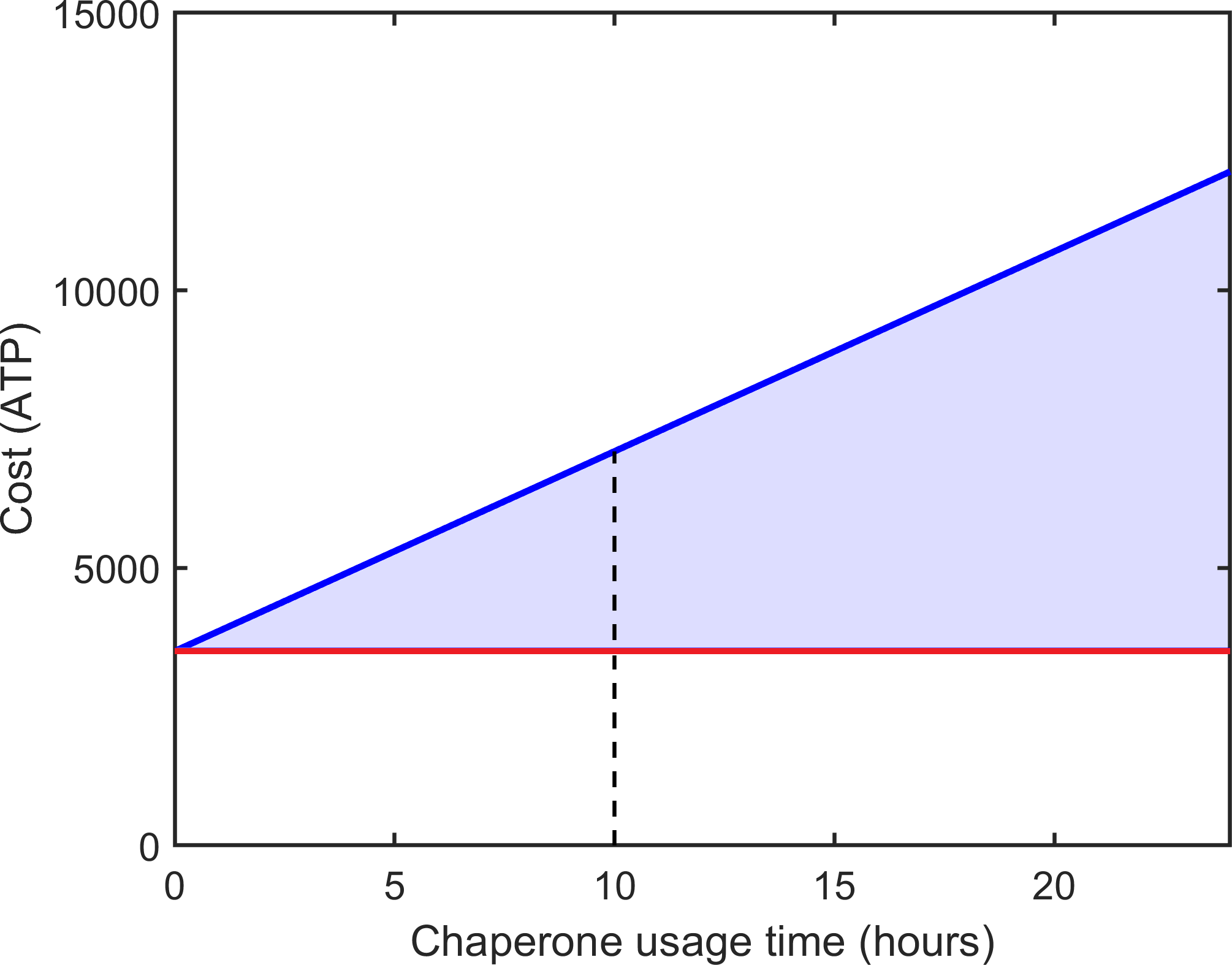

Supplement: S2 Fig — Total foldase cost (blue line) is the sum of an initial synthesis cost and an increasing “running cost” (shaded area) whereas holdases do not consume ATP and have a flat synthesis cost (red line). The running cost of an HSP70-type foldase in constant use is estimated to equal the synthesis (polymerization) cost after roughly 10 hours (dashed line). This stretches to roughly 100 hours for HSP60-class chaperones due to cycle times that are an order of magnitude slower (ATP consumption per kDa per cycle is comparable to HSP70 chaperones) (see S4 Fig). (TIF) [file pcbi.1008460.s002.tif]

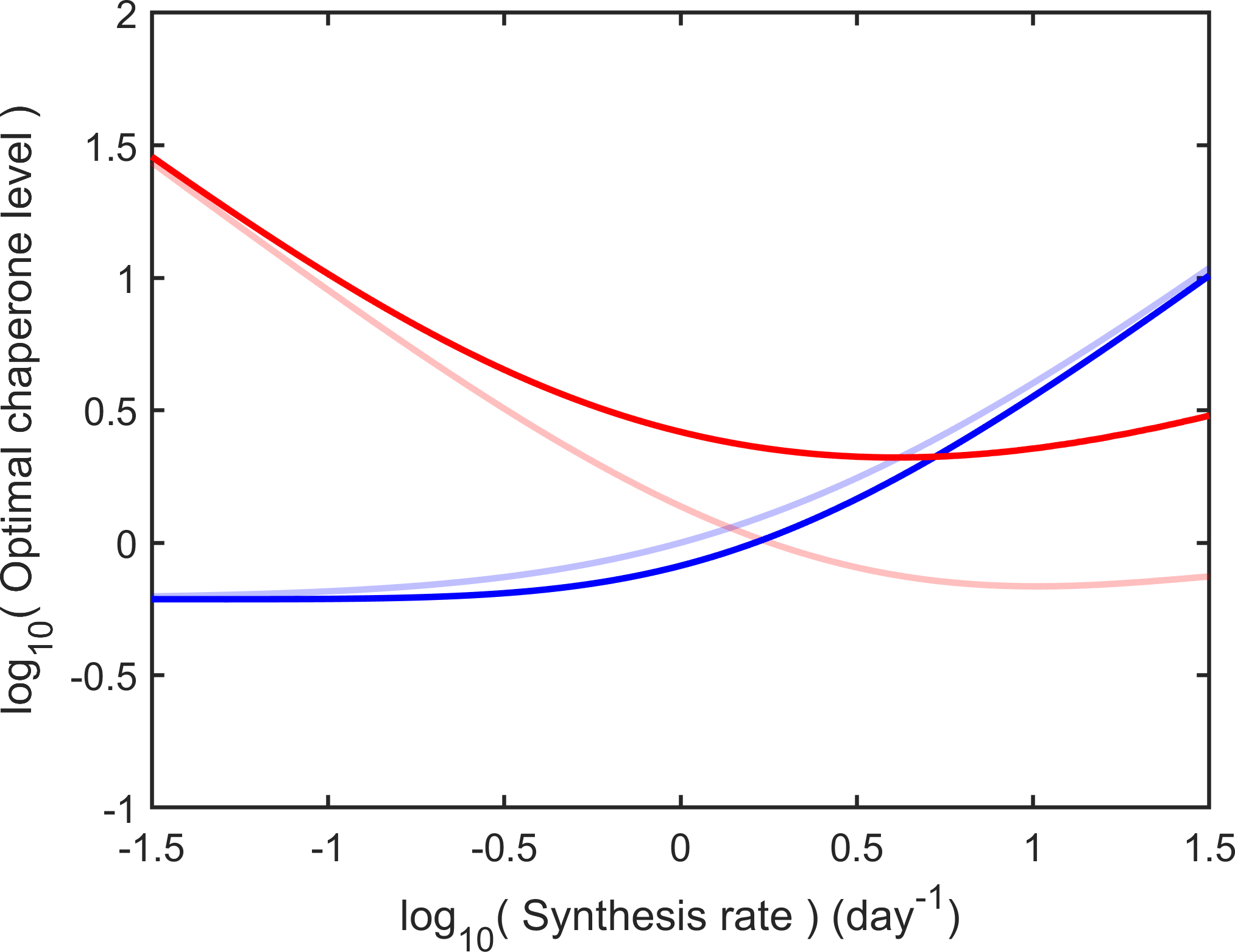

Supplement: S3 Fig — Effect on optimal chaperone expression if foldase size or synthesis cost is increased 10-fold from 70 kDa (3,500 ATP) to 700 kDa (35,000 ATP). (TIF) [file pcbi.1008460.s003.tif]

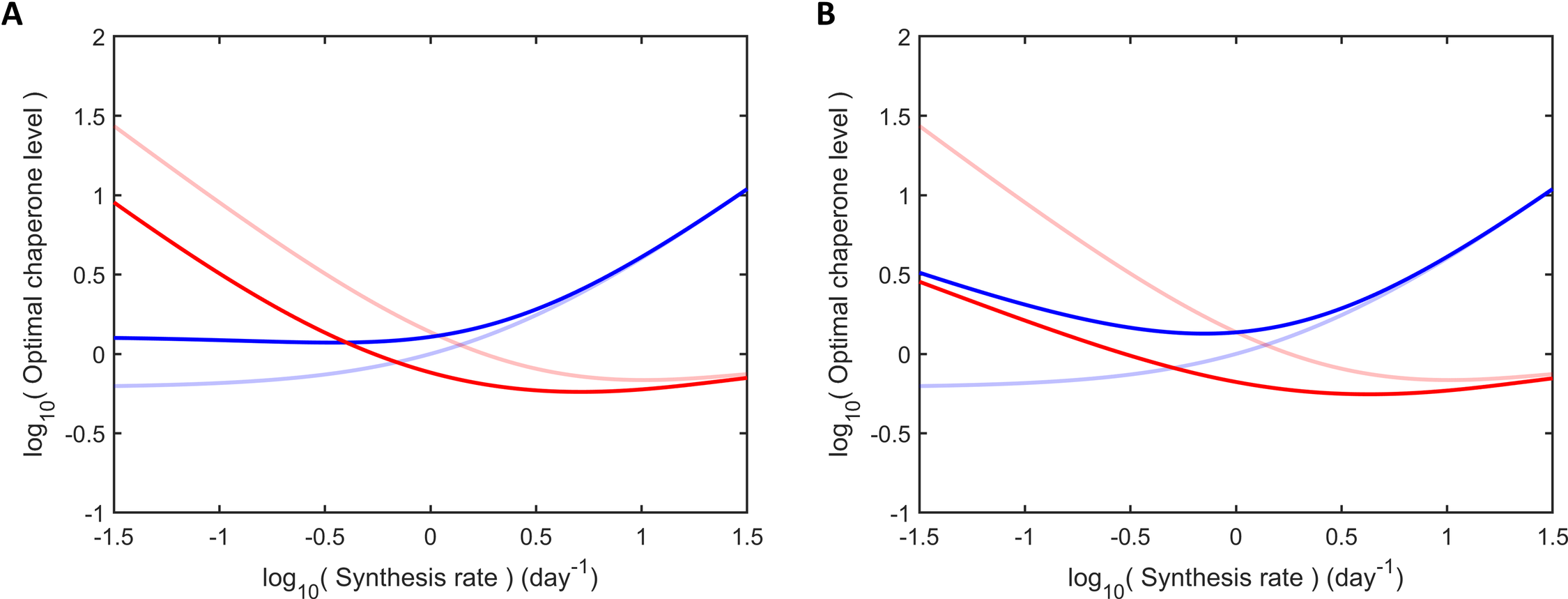

Supplement: S4 Fig — (A) Effect on optimal chaperone expression if foldase activity goes from “always on” to 10% ATPase activity when not folding proteins (ATP-dependent chaperones have functions other than folding). (B) In the limit of “perfect foldases” that have zero ATPase activity when not folding proteins, foldases “split the difference” with holdases, each scaling as 1/ksyn. (TIF) [file pcbi.1008460.s004.tif]

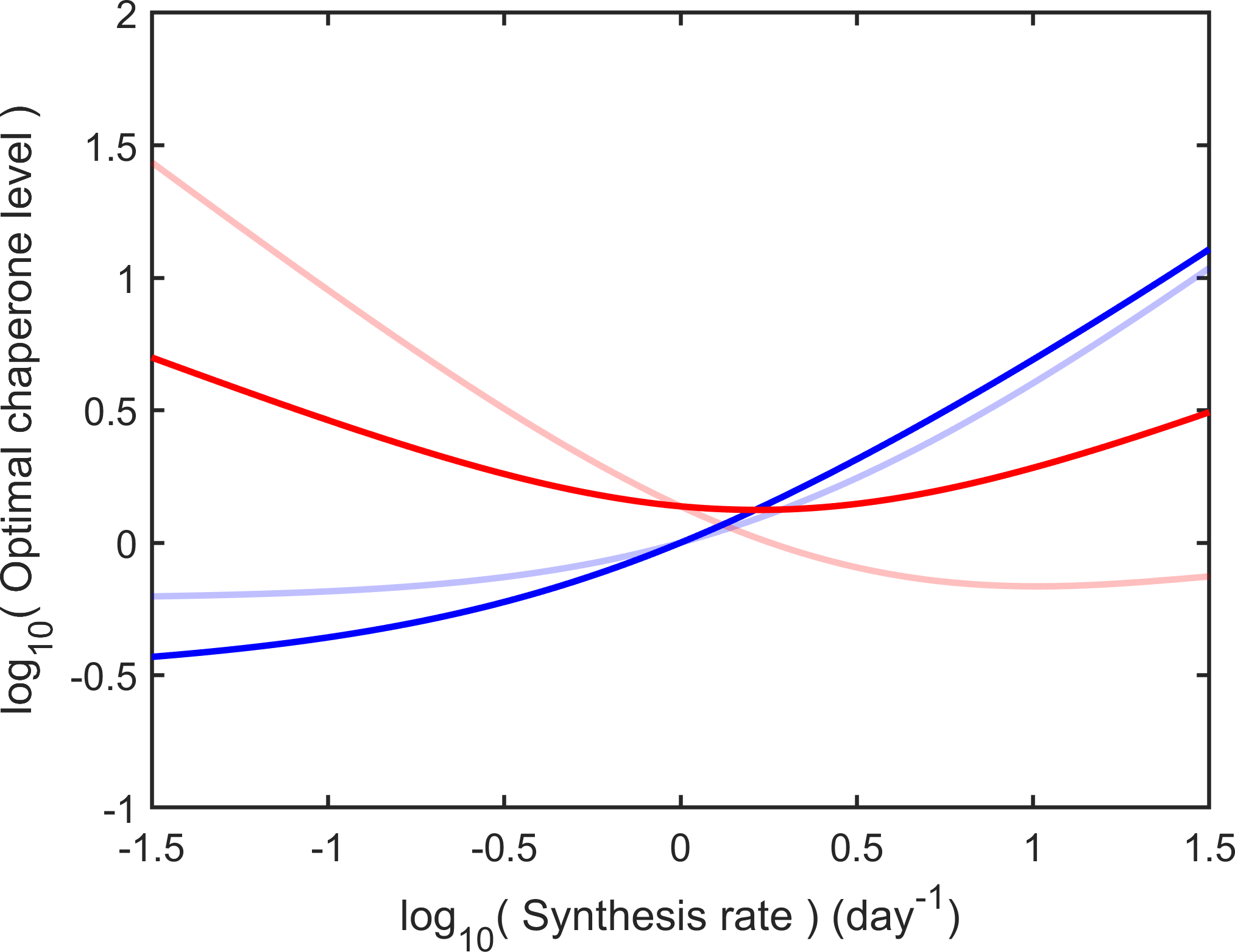

Supplement: S5 Fig — Effect on optimal chaperone expression if unfolding requirement goes from [Ufree] = uoksyn to [Ufree] = 100 nM, where uo = 100 nM∙day (see S1 Table). (TIF) [file pcbi.1008460.s005.tif]

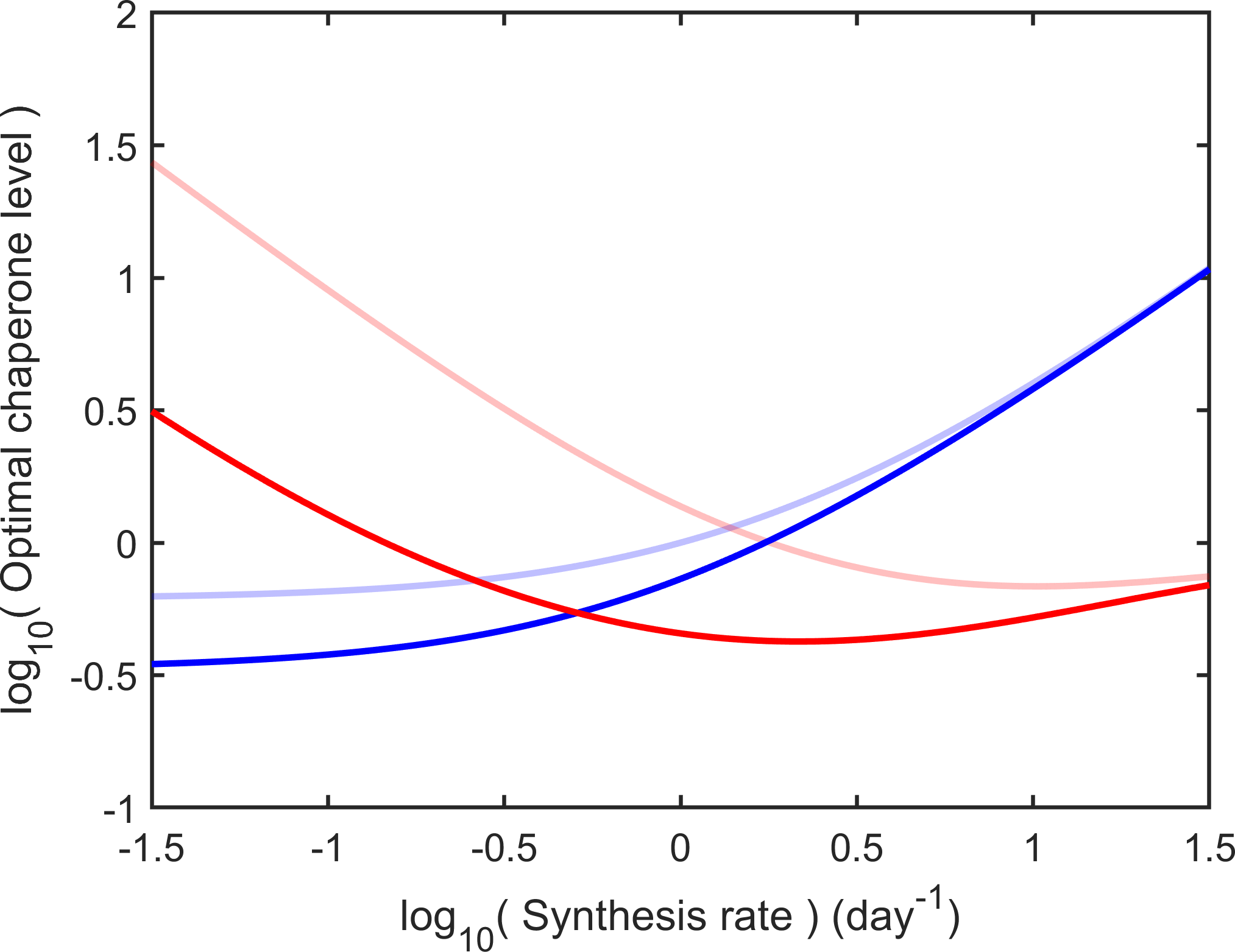

Supplement: S6 Fig — Effect on optimal chaperone expression from holdases transitioning from a loose binding (Kd = 1 μM; light curves) to tight binding regime (Kd = 10 nM; dark curves). Holdases (red) and foldases (blue). (TIF) [file pcbi.1008460.s006.tif]
