# Supplementary material for: Proteostasis is adaptive: Balancing chaperone holdases against foldases
Source: PLoS Comput Biol. 2020 Dec 14;16(12):e1008460. doi: 10.1371/journal.pcbi.1008460 (PMC7769611; doi:10.1371/journal.pcbi.1008460)
Supplement: S1 Text — (DOCX) [file pcbi.1008460.s007.docx]

**Proteostasis is adaptive: balancing chaperone holdases against foldases**

Adam MR de Graff, David E Mosedale, Tilly Sharp, Ken A Dill and David J Grainger

**Supporting Information Text**

**S1 Text. Dependence of optimal chaperone expression on chaperone properties**

A primary finding of this work is the disproportionate benefit (cost-effectiveness) of expressing more holdases at slow protein synthesis rates. These predictions depend on several key chaperone properties and their treatment in our simplified toy model. Here we explore these dependencies.

**Foldase running cost**

A key distinction between holdases and foldases is running cost. The model used in Figs 3 and 4 assumes foldases are constantly consuming ATP, meaning that greater foldase expression equates to greater total running cost. While this may seem a bit extreme, given the finite amount of protein that needs refolding, excess foldases could very well consume significant amounts of ATP through several actions. First, proteins possess many foldase binding regions and thus excess foldases could lead to binding of these other sites, causing less efficient folding. Second, foldases play non-folding roles that could become more active with excess foldase expression.

To explore the optimal chaperone expression under different scenarios, we reran the model assuming that foldases (i) have a small basal activity of 10% of the maximum while not bound to a client protein and (ii) are perfectly efficient when in excess, with no ATP consumed when not bound to a client. S3A Fig shows that the major conclusions remain unchanged even when excess foldases are largely turned off. Under the first scenario with 10% basal activity, foldases do become slightly more favorable at slow growth (at the expense of holdases), but the scaling behavior – ie. the slope with respect to synthesis rate – remains unchanged. However, in the limit of an ideal foldase, with no loss of efficiency from overexpression, the benefit of foldases is lost (S3B Fig). In this scenario, holdases and foldases “split the difference” and both rise with similar slopes. Interestingly, this appears to match the observed chaperone expression across rodent species (S2 Fig) and other mammals, albeit with variability between foldase types and tissues. While the cause of this variability currently remains unknown, higher organisms generally have much slower synthesis rates than *C. elegans* (Fig 3), perhaps placing a greater penalty on residual ATPase activity.

**Unfoldedness requirements**

The other major factor impacting model behavior is the requirement on [*U*_free_] at different synthesis rates. Based on the assumption that total unfolded dwell time should be constant, our model therefore assumes that [*U*_free_] scales with *k*_syn_. However, alternative scenarios could cause [*U*_free_] to have a weaker dependence on synthesis rate. This would occur if risks to unfolded protein decline under slower synthesis, such as from lower free radical levels, or if solubility is the key driver of loss. S5 Fig shows that the model prediction under the scenario where [*U*_free_] is constant. While both holdases and foldases are needed in lower abundance at low growth rates, the major conclusions remain intact. Similar robustness was found to changes in holdase dissociation constant, as occur during transitions from weak to tight binding modes, as well as to changes in chaperone mass.
