# Supplementary material for: Proteostasis is adaptive: Balancing chaperone holdases against foldases
Source: PLoS Comput Biol. 2020 Dec 14;16(12):e1008460. doi: 10.1371/journal.pcbi.1008460 (PMC7769611; doi:10.1371/journal.pcbi.1008460)
Supplement: S1 Table — (DOCX) [file pcbi.1008460.s008.docx]

**Proteostasis is adaptive: balancing chaperone holdases against foldases**

Adam MR de Graff, David E Mosedale, Tilly Sharp, Ken A Dill and David J Grainger

**Supporting Information Table**

**S1 Table.** **Parameters of the model.**

| Parameter | Description | Value | Reference |
| --- | --- | --- | --- |
| *k*_N→U_ | Unfolding rate | 1 day^-1^ | [1] |
| *n*_cyc_ | Foldase cycles to fold | 30 | (1] |
| *k*_foldase_ | Foldase bind-release cycle rate & ATP usage | 0.1 s^-1^ | [1] |
| *K*_m_ | Foldase Michaelis constant | 1 µM | [1] |
| *m*_foldase_ | Foldase molecular weight (HSP70) | 70 kDa | [2] |
| *K_d_* ^1^ | Holdase dissociation constant | 1 µM | [3] |
| *m*_holdase_ ^1^ | Holdase molecular weight (tetramer) | 70 kDa | [4] |
| *c*_syn,aa_ | Cost of synthesis | 5 ATP/aa | [5] |
| *P* | Total concentration of foldase-dependent protein | 1 mM | [1] |
| *u*_o_ ^2^ | Unfolding level coefficient | 0.1 µM∙day | Estimated |

^1^ Holdases vary in size and dissociation constant. Here we take holdases to be functioning in a low affinity mode where maximum substrate binding capacity has been estimated to be one client per four holdase monomers. The combined mass of four monomers is comparable to that of HSP70.

^2^ Free unfolded levels estimated to be on the order of 0.1 µM for a synthesis rate of 1 day^-1^ (see Fig. S5).

**References**

1. Santra M, Dill KA, De Graff AMR. Proteostasis collapse is a driver of cell aging and death. Proc Natl Acad Sci U S A. 2019;116(44):22173–8.

2. Hartl FU, Bracher A, Hayer-Hartl M. Molecular chaperones in protein folding and proteostasis. Nature. 2011;475:324–32.

3. Hilton GR, Hochberg GKA, Laganowsky A, Mcginnigle SI, Baldwin AJ, Benesch JLP, et al. C-terminal interactions mediate the quaternary dynamics of a B-crystallin. Philos Trans R Soc B Biol Sci. 2013;368:20110405.

4. Ehrnsperger M, Gräber S, Gaestel M, Buchner J. Binding of non-native protein to Hsp25 during heat shock creates a reservoir of folding intermediates for reactivation. EMBO J. 1997;16(2):221–9.

5. Amthor JS. The McCree-de Wit-Penning de Vries-Thornley respiration paradigms: 30 Years later. Ann Bot. 2000;86(1):1–20.
