# Supplementary material for: Proteostasis is adaptive: Balancing chaperone holdases against foldases
Source: PLoS Comput Biol. 2020 Dec 14;16(12):e1008460. doi: 10.1371/journal.pcbi.1008460 (PMC7769611; doi:10.1371/journal.pcbi.1008460)
Supplement: Attachment — Submitted filename: responseToReviewers.docx [file pcbi.1008460.s011.docx]

Dear Editor,

Corrections and additions were made to the manuscript to address the revisions requested by the reviewers. In addition, the files needed to run the model and reproduce all data have been included. This has led to the creation of Table S2 containing a summary of expression data for all known ATP-dependent chaperone foldases and ATP-independent holdases that I hope will be of help to other researchers, including some that I have communicated with in the process of writing this manuscript. Of the reviewers, only Reviewer 3 recommended revisions. Responses to their comments and changes made are described below in bold and highlighted in yellow in the revised manuscript. I greatly appreciate your help and patience.

Best regards,

Adam de Graff

Response to reviewer comments:

**I greatly appreciate the reviewers’ comments to help improve this paper. Only Reviewer 3 recommended changes. I have addressed all their comments, including correcting the noted typos in the main text and titles of references. Summaries of how I addressed the other comments can be found in bold below.**

Comment 1:

*• I’m a little confused by the red and blue lines in Figs 4a and b. Are they simply the model predictions from Fig 3 shifted on the y-axis such that day 1 = 0? I feel this could be made clearer in the text.*

**Yes, the curves are the model predictions relative to day 1 (ie. shifted such that day 1 = 0). This is now made clear in the main text as well as included on the axis labels of the plot. Figures 3 and 4C have been similarly shifted to day 1 = 0.**

Comment 2:

*• Perhaps some remarks could be made about the completeness of the data in Figs 4a and b. Are all known holdases/foldases included? If not, why were the selected examples chosen.*
**Yes, all known holdases and foldases with measured age-dependent expressions are included on the plot. This is now stated in the main text.**

Comment 3:

*• Can anything be stated about the substantial range in the data used for Fig 4a, i.e. why is hsp-17 level seemingly independent of Ksyn?*

**I have now added sentences describing the range of slopes seen among holdases, as well as adding one holdase that I found when revising the manuscript (it lacks a gene name and is not well studied). I describe possible reasons for the deviation by sip-1 (to the upside). In contrast, little is known about hsp-17 in *C. elegans*, but this, along with its deviation from the prediction, is now stated in the main text.**

*• It is stated in the text that the foldase least-squares fit in 6A is “highly dependent on the outlier”, yet it seems from the figure legend that the outlier has actually been excluded from the fit. This should be checked and clarified. Also, the outlier should be marked somehow on the graph (e.g. different symbol)*. **The slopes with and without the outlier are now described, along with the R^2^ of the respective fits. The foldase data in (A) is now HSC70+HSP70 (ie. the constitutive and induced form of HSP70) and noted in the text.**

*• The goodness of fit (R^2 or similar) of the least-squares fits used in Figure 6 should be reported.*
**The R^2^ of the least-squares fits have now been reported.**

*• It seems that the foldase data in Fig 6A might better be described by the “perfect foldase” model in Fig S4. Could it be that foldase efficiency is different in younger organisms, explaining the disparity between the model and experimental data for foldase levels vs metabolic rate in 6A?*

**I agree with this great observation that, indeed, the data is in the direction of the “perfect foldase”. I have expanded greatly on this in the main text, adding two additional paragraphs discussing implications (highlighted yellow).**

**Additional changes:**

**While the section on inter-species differences in foldase expression has been expanded, I have removed Figure 5 that displayed changes in foldase *client protein* abundance with age. I believe this is appropriate for several reasons: (1) It provided indirect evidence for the paper’s main hypothesis, as declining client protein abundances could have alternative explanations (such as altered mRNA transcription, differential expression of entire organelles such as mitochondria late in life or enhanced clearance). (2) Discussion of these alternative possible causes for altered client levels deserves separate consideration that is tangential to this paper’s main point. A paper should have one message. This paper’s message is that the holdase-foldase balance is dictated by a minimum energy principle, which we support with available data on holdase and foldase expression. I have expanded the Discussion to bring focus to this message.**

**Reproducibility:**

**I have now included all files needed to run the model and generate all the data and figures in this manuscript, including comments and instructions within the files.**

**Thanks again to the reviewers for their help to improve this paper.**

**Best regards,**

**Adam de Graff**
